# Supplementary material for: MDA5 generates compact ribonucleoprotein complexes via ATP-dependent double-stranded RNA unwinding
Source: Nucleic Acids Res. 2026 Mar 31;54(6):gkag274. doi: 10.1093/nar/gkag274 (PMC13036495; doi:10.1093/nar/gkag274)
Supplement: gkag274_Supplemental_Files [file gkag274_supplemental_files.zip › Quack et al_SI_NAR_revised.pdf]

Supplementary Materials for

MDA5 generates compact ribonucleoprotein complexes via ATP-dependent double-stranded RNA unwinding

Salina Quack *et al.*

*Corresponding author. Email: d.dulin@vu.nl

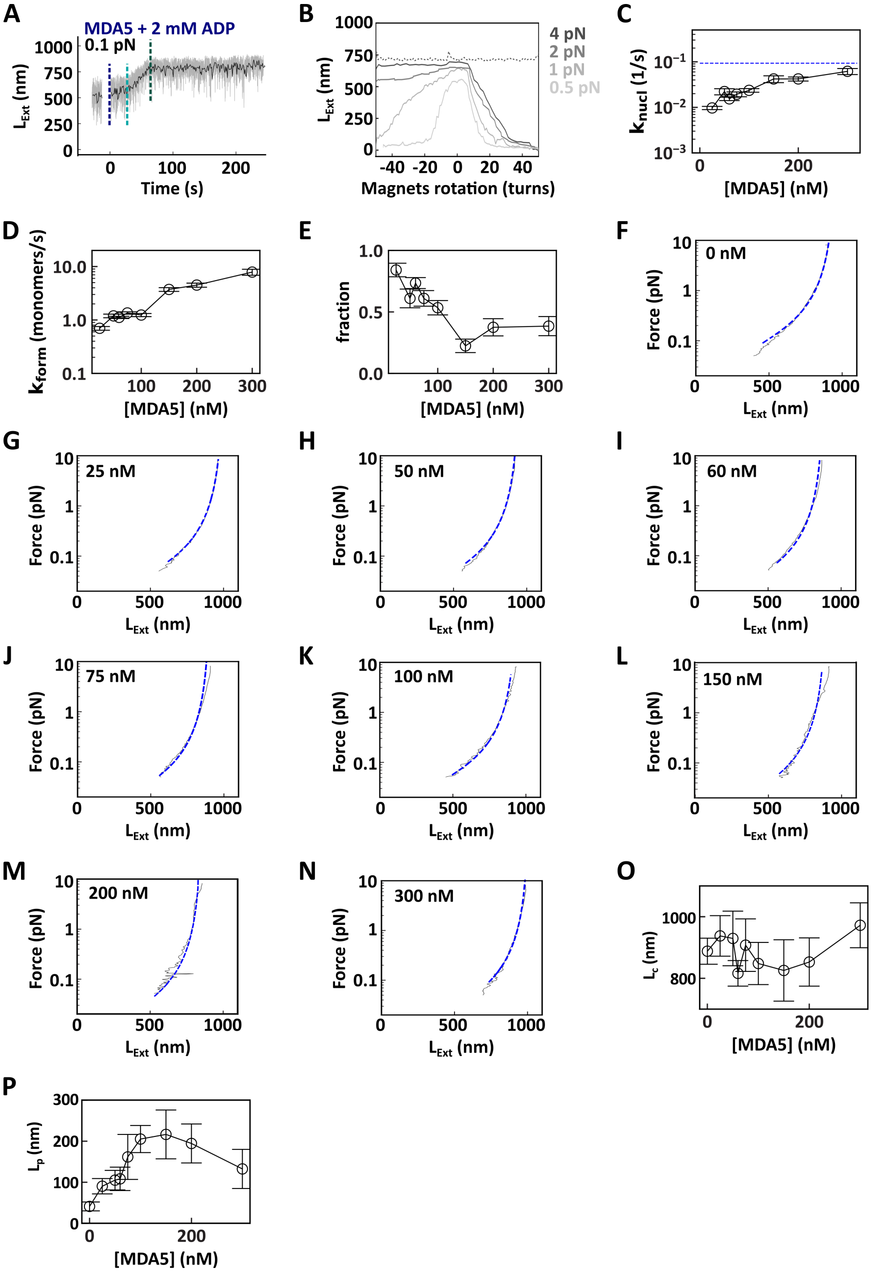

Figure S1: Force-extension traces of MDA5 filaments at different MDA5 concentrations. (A) Filament formation trace of 100 nM MDA5 and 2 mM ADP at 0.1 pN experimental conditions. The raw (58 Hz) and time averaged (1 Hz) traces are represented in grey and black, respectively. The vertical dashed lines indicate the end of each phase: flushing of MDA5 with ADP (dark blue), nucleation (teal), and filament formation (green). **(B)** Rotation-extension traces of non-coilable dsRNA (dotted line) and coilable dsRNA (solid lines) at various forces. **(C)** Mean nucleation rate of MDA5 on dsRNA in the presence of AMP-PNP. The blue dotted line indicates the limit for the nucleation rate based on the time it takes to add the proteins to the flow cell. **(D)** Mean filament formation rate on dsRNA in the presence of AMP-PNP. **(E)** Relative fraction of nucleation events that were longer than the flush duration. **(F- N)** Mean force extension (black line) and a fit using the worm-like chain (WLC) model (blue dashed line, **Equation 1**). Force extension of **(F)** ~3.2 kbp dsRNA and MDA5 filaments formed using either **(G)** 25 nM, **(H)** 50 nM, **(I)** 60 nM, **(J)** 75 nM, **(K)** 100 nM, **(L)** 150 nM, **(M)** 200 nM, or **(N)** 300 nM WT MDA5, respectively, in the presence of AMP-PNP. Statistics and WLC model fits parameters values are provided in **Table S2**. **(O)** Mean contour length and **(P)** persistence length of MDA5 filaments. The error bars are one standard deviation. (C, D, O, P) Error bars represent the standard deviation from 1000 bootstraps. The lines are guides for the eye. Statistics, mean, and error values are provided in **Table S2** and **Table S3**.

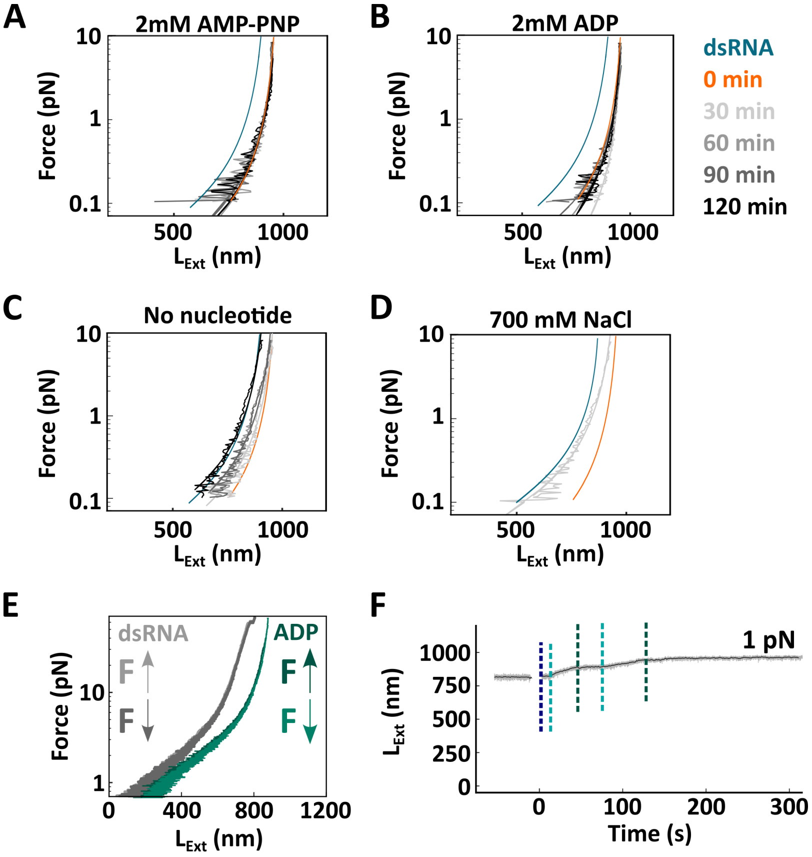

Figure S2: MDA5 filament stability in different conditions, pauses in filament assembly and assembly kinetics on a coilable dsRNA tether. Mean force extension of MDA5 filament in the presence of either **(A)** 2 mM AMP-PNP or **(B)** 2 mM ADP, **(C)** formed in absence nucleotide, or **(D)** after treatment with the standard reaction buffer containing 700 mM NaCl in comparison to bare dsRNA (blue) and a MDA5 filament at t=0 min (orange). Color code described on the right-hand side of panel (B). Statistics, mean, and error values from the WLC fits are provided in **Table S4**. **(E)** Force-extension of dsRNA (grey) and MDA5 filament (green) in the presence of ADP upon dynamically increasing (light) and decreasing force (dark). **(F)** Filament formation trace of MDA5 in the presence of AMP-PNP on a non-coilable dsRNA tether at 1 pN.

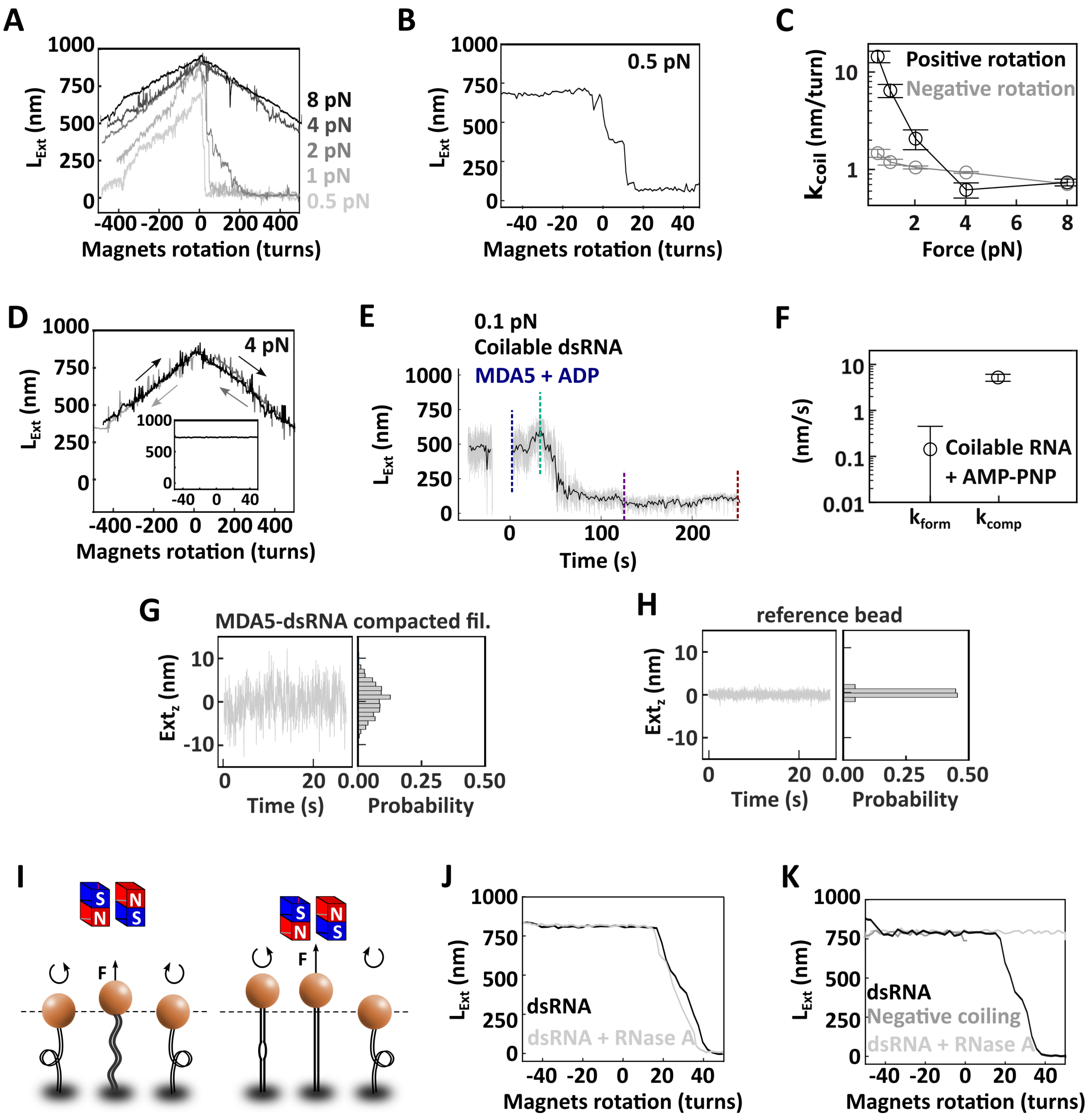

Figure S3: Torsional behavior of the MDA5-dsRNA filament. (A) Rotation extension of 100 nM MDA5-dsRNA filaments in presence of 2 mM ADP at 0.5 pN (light grey), 1 pN (grey), 2 pN (medium grey), 4 pN (dark grey), and 8 pN (black). **(B)** Rotation-extension trace of 100 nM MDA5 filament in the presence of 2 mM ADP between -50 and 50 turns. **(C)** Mean decrease in extension rate from data in (F) for either negative (grey) or positive (black) turn addition. The error bars are the standard deviation from 1000 bootstrap samples. The lines are guides for the eye. Statistics, mean, and error values are provided in **Table S5**. **(D)** Rotation-extension of a non-coilable dsRNA tether after nucleoprotein filament formation with 100 nM MDA5 and 2 mM ADP. The arrows indicate the direction of rotations applied. Insert: Rotation extension trace of the tether at 4 pN before MDA5 injection in the flow chamber. **(E)** Time trace of a filament assembly on a coilable dsRNA. The vertical dashed lines indicate the end of each phase: flushing of 100 nM MDA5 with 2 mM ADP (blue), respectively, nucleation (teal), filament formation (green), filament compaction (purple) and the compacted filament (red). The raw (58 Hz) and time averaged (1 Hz) traces are represented in grey and black, respectively. **(F)** Mean formation and compaction rates of MDA5 filament on a coilable dsRNA tether. Error bars represent the standard deviation from 1000 bootstrap samples. Statistics, mean, and error values are provided in **Table S7. (G)** Time trace of an MDA5-dsRNA filament after compaction with probability histogram of the fluctuations in the z-extension. **(H)** Time trace of a reference bead. with probability histogram of the fluctuations in the z-extension. (G, H) The raw (58 Hz) and time averaged (1 Hz) traces are represented in grey and black, respectively. **(I)** Description of a rotation-extension experiment of a coilable dsRNA tether at low and high force (left and right, respectively). **(J)** Rotation-extension trace of a coilable dsRNA tether at 4 pN before (black) and after (light grey) the addition of 10 ng RNase A while the tether was torsionally relaxed and at 4 pN. **(K)** Rotation-extension trace of the same dsRNA tether at 4 pN before (black) and after (light grey) the addition of 10 ng RNase A while the tether was negatively supercoiled and at 4 pN.

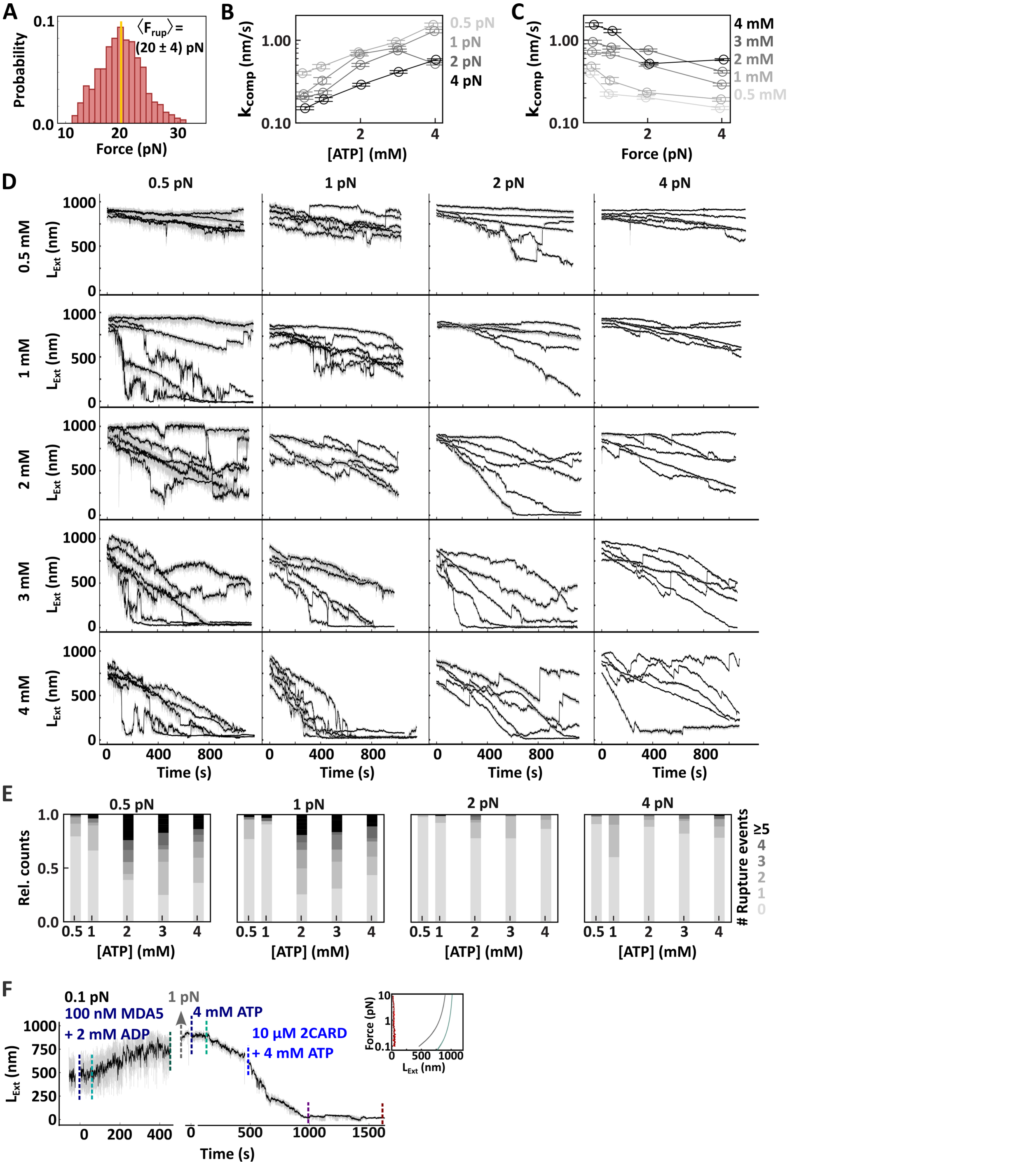

Figure S4: MDA5 filament compaction is force and ATP concentration dependent. (A) Distribution of rupture forces observed when breaking the oligomerized filament after ATP hydrolysis. Mean rupture force indicated in yellow and above the plot (the error is one standard deviation). **(B, C)** Mean compaction rate of MDA5-dsRNA nucleoprotein filaments for the **(B)** force or **(C)** ATP concentration indicated above. **(D)** Time traces of filament compaction in the presence of the indicated concentration of ATP and force. The raw (58 Hz) and time averaged (1 Hz) traces are represented in grey and black, respectively. **(E)** Relative counts of rupture events for filament compaction experiments as shown in (A). **(F)** Time trace of 100 nM MDA5 filament formation in presence of 4 mM ATP at 1 pN. 10 µM 2CARD with 4 mM ATP was added to the compacting filament (blue dashed line). Small insert: Force-extension curve of the compacted filament (red lines) at the end of the experiment. Non-extensible WLC fits to force-extension experiments for dsRNA (grey line) and MDA5 filament (teal line). (B, C, E) Statistics, mean, and error values are provided in **Table S7**.

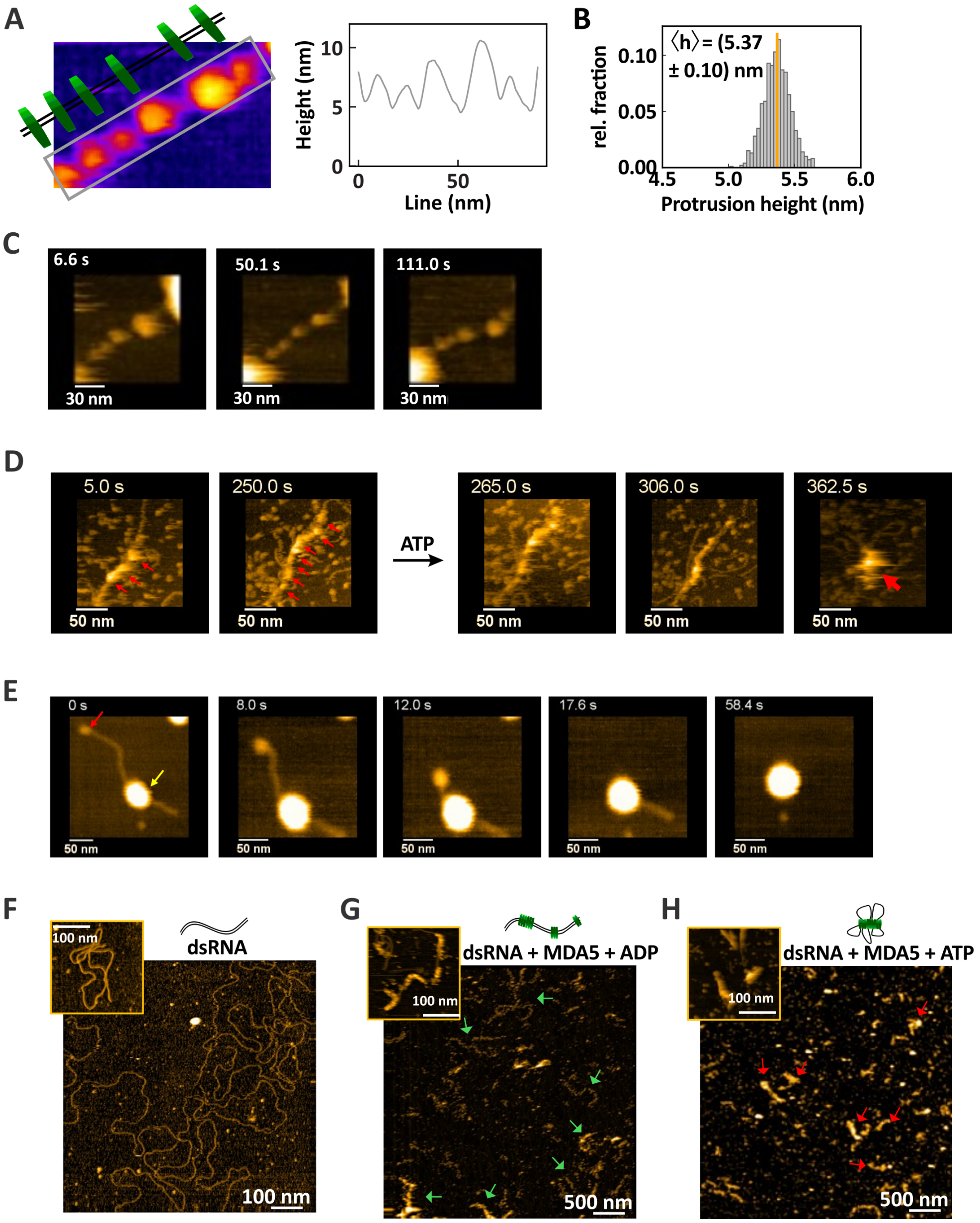

Figure S5: High-speed AFM images of a MDA5-dsRNA nucleoprotein filament. (A) AFM image of dsRNA-MDA5 complex in presence of 2mM ADP (left) and schematic of the MDA5-dsRNA filament. Height profile (right) along a line cross section through the middle of the grey rectangle (left). **(B)** Distribution of the protrusion height of MDA5 monomers bound to dsRNA in presence of ADP (n=47). The distribution, mean and std were calculated from 1000 bootstrap samples. The mean is indicated in orange. **(C)** Snapshot HS-AFM images from Video S3 of the dsRNA-MDA5 complex in the presence of 2 mM ADP imaged over time. The imaging rate is 300 milliseconds per frame. **(D)** Snapshot HS-AFM image of Video S4 of dsRNA-MDA5 complex in the presence of 1 mM ADP and 10 mM ATP. The red arrows indicate the MDA5 on RNA. Imaging speed is 2 frames/second. **(E)** Snapshot HS-AFM image of Video S5 of dsRNA-MDA5 complex in the presence of 10 mM ATP. The red arrow indicates the position of the MDA5 on the dsRNA, and the yellow arrow localises random aggregates on the surface. Imaging speed is 1 frame/second. **(F)** HS-AFM image of 20 nM dsRNA attached to the mica surface. Inset showing single dsRNA on mica. **(G)** HS-AFM image of 20 nM dsRNA incubated in 50 nM MDA5 in the presence of 1 mM ADP and 5 mM MgCl2 in solution and later immobilised on Mica. Green arrows indicate the dsRNA-MDA5 complex. The inset shows a zoomed image of the complex. **(H)** Same as (G), but after the addition of 10 mM ATP in the imaging chamber. Red arrows indicate examples of the compacted structure of the dsRNA-MDA5 complex. The inset shows a magnified image of the RNA-MDA5 structure after compaction.

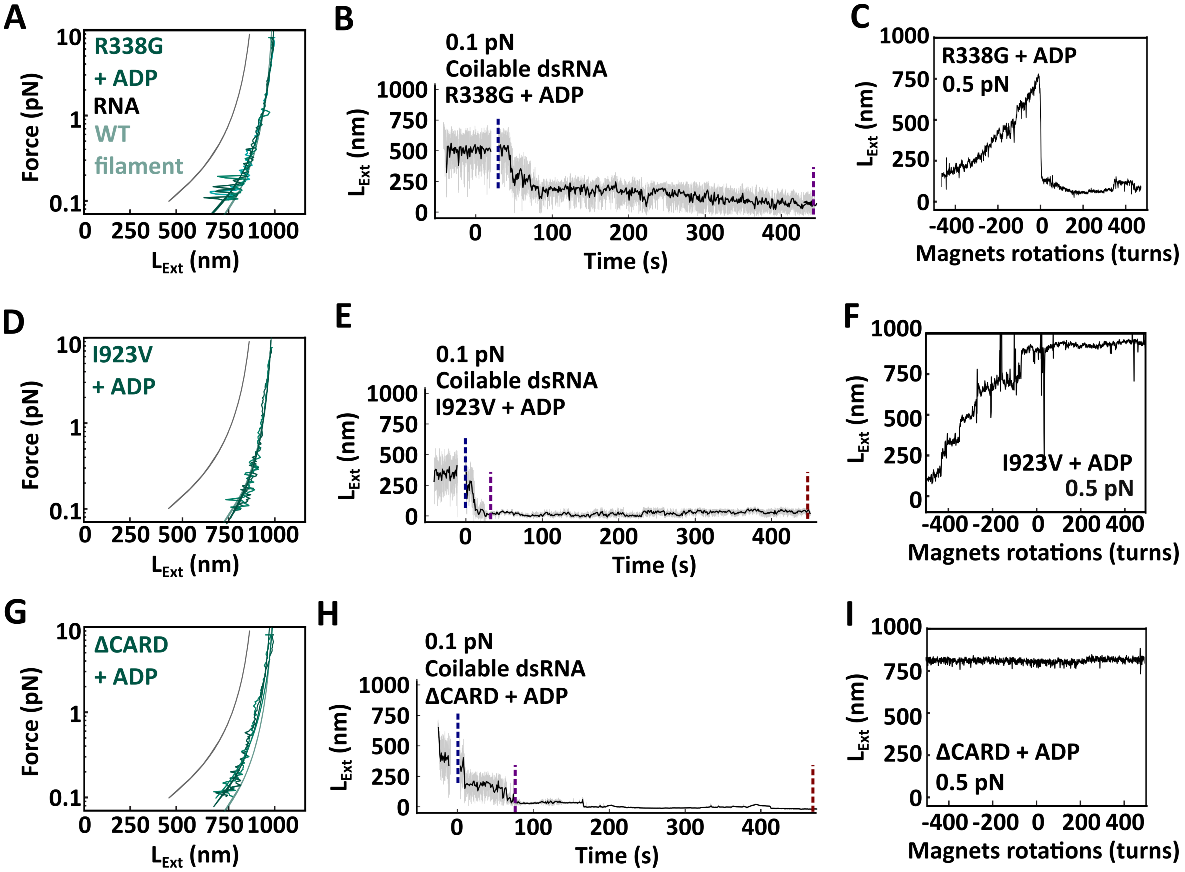

Figure S6: Filament formation with either MDA5 ΔCARD or MDA5 R338G is similar to WT MDA5. (A) Force-extension traces of MDA5 R338G filaments formed with ADP (green). **(B)** Time trace filament formation on a coilable dsRNA tether with 100 nM MDA5 R338G and 2 mM ADP. **(C)** Rotation-extension trace of MDA5 R338G filament assembled with ADP**. (D)** Force-extension traces of MDA5 I923V filaments formed with ADP (green). **(E)** Time trace of filament formation on a coilable dsRNA tether with 100 nM MDA5 I923V and 2 mM ADP. **(F)** Rotation-extension of MDA5 I923V filament. **(G)** Force-extension traces of MDA5 ΔCARD filaments formed with ADP (green). **(H)** Time trace of filament assembly in the presence of ADP on a coilable dsRNA tether with 100 nM MDA5 ΔCARD and 2 mM ADP. **(I)** Rotation-extension trace of MDA5 ΔCARD filament assembled with ADP**.** (A, D, G) The mean force-extension traces of dsRNA (grey line) and WT MDA5 filament (teal line) are represented for comparison. Statistics and non-extensible WLC fit parameters are provided in **Table S8**. (B, E, H) The raw (58 Hz) and time averaged (1 Hz) traces are represented in grey and black, respectively.

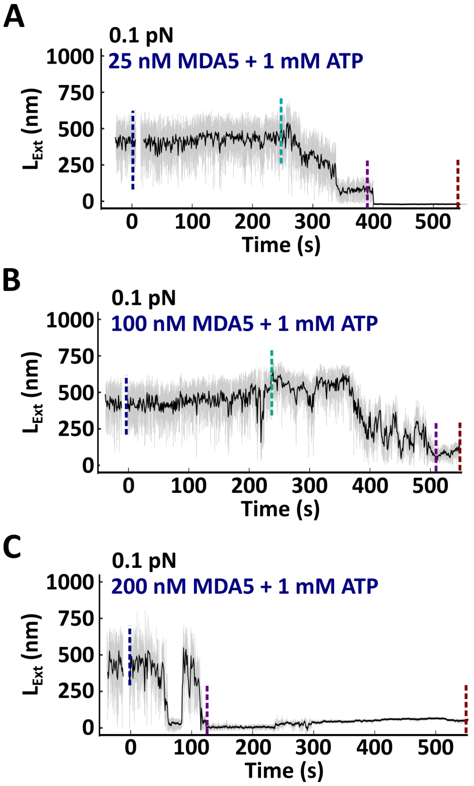

Figure S7: Assay resolution and filament nucleation, formation and compaction rates as a function of MDA5 concentration in presence of ATP. (A, B, C) Time traces of MDA5 filament formation in presence of 1 mM ATP and 0.1 pN with **(A)** 25 nM, **(B)** 100 nM, or **(C)** 200 nM MDA5, respectively. The vertical dashed lines indicate the end of each phase: flushing (dark blue), pre-compaction (teal), compaction (purple) and the compacted filament (red). The raw (58 Hz) and time averaged (1 Hz) traces are represented in grey and black, respectively.

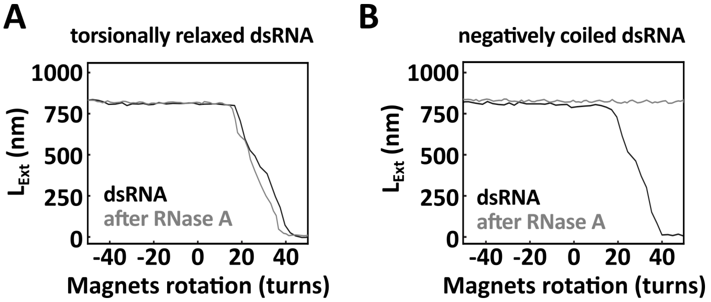

Figure S8: RNase A treatment of dsRNA (A) Rotation extension curve of torsionally relaxed dsRNA before (black) and after (grey) RNase A addition at 1 pN. RNase A was added and washed after ~10s with ~500 µl PBS. **(B)** Rotation extension curve of negatively supercoiled dsRNA before (black) and after (grey) RNase A addition at 1 pN. RNase A was added and washed after ~10s with ~500 µl PBS.

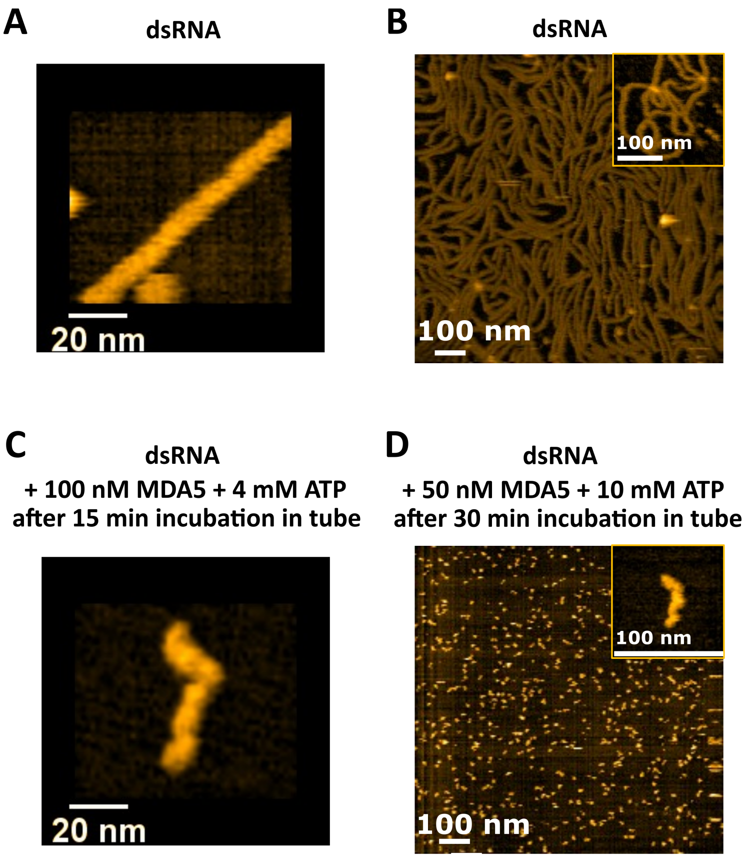

Figure S9: High-speed AFM images of bare dsRNA and ssRNA. (A) HS-AFM images taken for a bare dsRNA on mica. **(B)** Same as (A) but of a larger area. Inset shows single dsRNA on mica. **(C)** HS-AFM image of a sample of 100 nM MDA5 with 4 mM ATP and dsRNA after incubation for 15 min in a reaction tube. Only ssRNA strands remained present in the sample. **(D)** HS-AFM image of 100 nM dsRNA incubated for 30 minutes in 50 nM MDA5 in the presence of 10 mM ATP + 10 mM MgCl2 in solution and later immobilised on mica. The inset shows a magnified image of the single ssRNA on mica.

Video S1: HS-AFM movie capturing the MDA5 –dsRNA interaction in presence of 2 mM ADP in real-time. The imaging rate is 300 milliseconds per frame.

Video S2: HS-AFM movie capturing the MDA5 –dsRNA interaction in presence of 2 mM ADP in real-time. The imaging rate is 300 milliseconds per frame.

Video S3: HS-AFM movie capturing the MDA5 –dsRNA interaction upon 2 mM ADP and 6 mM ATP hydrolysis in real-time. The imaging rate is 500 milliseconds per frame.

Video S4: HS-AFM movie capturing the MDA5 –dsRNA interaction upon 1 mM ADP and 10 mM ATP hydrolysis in real-time. The imaging rate is 300 milliseconds per frame.

Video S5: HS-AFM movie capturing the MDA5 –dsRNA interaction upon 10 mM ATP hydrolysis in real-time. The imaging rate is 100 milliseconds per frame.

Video S6: This is an example HS-AFM movie capturing the ssRNA-like coiled structure on mica extracted from a mixture of dsRNA, 100 nM MDA5, and 4 mM ATP. The imaging rate is 500 milliseconds per frame.

Table S1: Primers for RNA constructs

5'-3' strand BIO handle fw	GGATCCTACCTGACGCTTTT
5'-3' strand BIO handle rev	TAATACGACTCACTATAGGCAAACGGCTTGATATCC
5'-3' strand strand fw	ATTCAGGGACTGCCGATG
5'-3' strand strand rev	TAATACGACTCACTATAGGACGTTTCGGATCTTCC
5'-3' strand DIG handle fw	GGAGCGTAAAATTCAGTTCTTC
5'-3' strand DIG handle rev	TAATACGACTCACTATAGGTTAACCTCAACTTCCATTTCC
5'-3' strand 3'-gap up fw	TCGTGTCACCCAGTCGGACC
5'-3' strand 3'-gap up rev	TAATACGACTCACTATAGGAGCGCCGCTTCCATGTCCTGGAACGCT
5'-3' strand 5'-gap down fw	TAATACGACTCACTATAGGCACGTCGCGCAGATG
3'-5' strand 3'-gap down fw	TAATACGACTCACTATAGGCGGATAACGAACATCTG
3'-5' strand 3'-gap down rev	TCCGACATGGAACGCAA
3'-5' strand rev	GGTTAACCTCAACTTCCATTTCC
3'-5' strand fw	TAATACGACTCACTATAGGATCCTACCTGACGCTTTT
3'-5' strand 5'-gap up fw	TAATACGACTCACTATAGGCGCGCGTGCTGC
3'-5' strand 5'-gap up rev	TGTGTCGGCTGCACCGAC
3'-5' strand DIG handle for 3'-gap up fw	TAATACGACTCACTATAGGTCGGATAAGGCGTTAGG
3'-5' strand 3'-gap up rev	ATGGAACGCAAATCATCAGC

Table S2: Mean filament nucleation and formation rate of MDA5-dsRNA filament in presence of 2 mM AMP-PNP at 0.1 pN

[MDA5] (nM)	k_{nucl}	±	std (1/s)	k_{form}	±	std (monomers/s)	k_{form}	±	std (nm/s)	N
25	0.010	±	0.001	0.7	±	0.1	0.8	±	0.1	44
50	0.022	±	0.003	1.2	±	0.1	1.4	±	0.1	34
60	0.015	±	0.001	1.1	±	0.1	1.2	±	0.1	98
75	0.019	±	0.002	1.4	±	0.1	1.6	±	0.1	55
100	0.024	±	0.002	1.2	±	0.1	1.5	±	0.1	70
150	0.042	±	0.007	3.9	±	0.3	4.3	±	0.3	53
200	0.042	±	0.004	4.5	±	0.4	5.0	±	0.4	42
300	0.062	±	0.009	7.9	±	1.0	8.5	±	1.1	33

Table S3: Mean contour length and persistence length of MDA5-dsRNA filaments in presence of 2 mM AMP-PNP

[MDA5] (nM)	L_C	±	std (nm)	L_p	±	std (nm)	N WLC
0	888	±	89	41	±	11	321
25	938	±	66	90	±	28	58
50	929	±	89	105	±	24	135
60	816	±	42	108	±	28	144
75	908	±	85	161	±	55	103
100	848	±	68	205	±	33	39
150	826	±	100	216	±	59	19
200	853	±	78	194	±	47	14
300	972	±	73	132	±	63	40

Table S4: Contour length and persistence length of MDA5-dsRNA filament after 30 min intervals

	30 min		60 min		90 min		120 min		N
	L_C ± std (nm)	L_P ± std (nm)	L_C ± std (nm)	L_P ± std (nm)	L_C ± std (nm)	L_P ± std (nm)	L_C ± std (nm)	L_P ± std (nm)	
2 mM AMP-PNP	929 ± 137	153 ± 87	914 ± 115	193 ± 93	911 ± 109	203 ± 93	916 ± 127	191 ± 81	16

2 mM ADP	998 ± 57	98 ± 92	961 ± 73	122 ± 11	1002 ± 56	171 ± 103	940 ± 73	168 ± 60	5
No nucleotide	965 ± 62	198 ± 147	939 ± 41	227 ± 86	933 ± 37	153 ± 42	933 ± 43	245 ± 143	2
700 mM NaCl	908 ± 94	56 ± 19	-	-	-	-	-	-	17

Table S5: Rotation rates of MDA5 WT-dsRNA filaments upon negative and positive supercoiling

Force (pN)	Negative k_{rot}	±	std (nm/turn)	N	Positive k_{rot}	±	std (nm/turn)	N
0.5	1.49	±	0.15	15	14.96	±	1.83	13
1	1.20	±	0.09	18	6.32	±	0.95	16
2	1.04	±	0.03	24	2.07	±	0.56	22
4	0.94	±	0.02	10	0.59	±	0.11	8
8	0.71	±	0.03	24	0.74	±	0.06	22

Table S6: Mean filament formation and compaction rate of MDA5-dsRNA filament in presence of 2 mM AMP-PNP at 0.1 pN on a coilable dsRNA tether.

[MDA5] (nM)	k_{form}	±	std (nm/s)	k_{comp}	±	std (nm/s)		N
100	0.1	±	0.3	5.2	±	0.9		6

Table S7: Compaction rates and reversal counts of MDA5 WT-dsRNA filaments

[ATP] (mM)	Force (pN)	k_{comp}	±	std (nm/s)	rel. count reversal events	±	std	N
1	0.1	1.35	±	0.19				67
0.5	0.5	0.40	±	0.02	0.4	±	0.8	102
1		0.48	±	0.03	0.7	±	1.5	77
2		0.72	±	0.04	2.7	±	2.9	55
3		0.95	±	0.05	2.2	±	2.3	52
4		1.53	±	0.08	1.8	±	2.2	95
0.5	1	0.22	±	0.01	0.4	±	1.0	44
1		0.32	±	0.03	0.3	±	1.1	33
2		0.68	±	0.03	2.4	±	2.7	59
3		0.81	±	0.03	2.0	±	2.3	81
4		1.29	±	0.06	1.7	±	2.3	64
0.5	2	0.20	±	0.01	0.0	±	0.1	46
1		0.23	±	0.01	0.2	±	0.8	91
2		0.50	±	0.02	0.4	±	0.8	41
3		0.76	±	0.03	0.3	±	0.5	68
4		0.52	±	0.02	0.2	±	0.5	84
0.5	4	0.15	±	0.01	0.1	±	0.5	57
1		0.19	±	0.01	0.5	±	0.7	43
2		0.29	±	0.01	0.2	±	0.6	62
3		0.41	±	0.01	0.3	±	0.7	51
4		0.58	±	0.01	0.4	±	1.1	163

Table S8: Mean persistence length and mean contour length of MDA5 mutant-dsRNA filaments

	$L_C \pm$ std (nm)	$L_P \pm$ std (nm)	N	$L_C \pm$ std (nm)	$L_P \pm$ std (nm)	N
	2 mM ADP			4 mM ATP		
R338G	886 ± 18	190 ± 18	66	865 ± 23	190 ± 20	66
I923V	885 ± 9	150 ± 23	177	-	-	
ΔCARD	945 ± 23	185 ± 19	68	886 ± 18	42 ± 2	68

Table S9: Trace classification of 50 nM MDA5 on gapped RNA in presence of 1 mM ATP at 1pN

RNA constructs	Extension			Dissociation			Partial Compaction			Full Compaction			Total
	Mean	±	SEM	Mean	±	SEM	Mean	±	SEM	Mean	±	SEM	N
dsRNA	5.6%	±	7.5%	0.0%	±	0.0%	27.8%	±	14.6%	66.7%	±	15.4%	36
3’-gap up	20.8%	±	16.2%	8.3%	±	11.1%	25.0%	±	17.3%	45.8%	±	19.9%	24
3’-gap down	31.0%	±	11.9%	3.4%	±	4.7%	36.2%	±	12.4%	29.3%	±	11.7%	58
3’-gap both	56.8%	±	16.0%	8.1%	±	8.8%	32.4%	±	15.1%	2.7%	±	5.2%	37
5’-gap down	25.0%	±	16.0%	7.1%	±	9.5%	21.4%	±	15.2%	46.4%	±	18.5%	28
5’-gap up	30.8%	±	10.2%	1.3%	±	2.5%	39.7%	±	10.9%	28.2%	±	10.0%	78
5’-gap both	8.5%	±	6.0%	28.0%	±	9.7%	19.5%	±	8.6%	43.9%	±	10.7%	82

Table S10: Trace classification of 400 nM MDA5 on gapped RNA in presence of 1 mM ATP at 1pN

RNA constructs	Extension			Dissociation			Compaction			Full Compaction			Total
	Mean	±	SEM	Mean	±	SEM	Mean	±	SEM	Mean	±	SEM	N
dsRNA	8.3%	±	11.1%	8.3%	±	11.1%	16.7%	±	14.9%	66.7%	±	18.9%	24
3’-gap up	21.4%	±	15.2%	3.6%	±	6.9%	57.1%	±	18.3%	17.9%	±	14.2%	28
3’-gap down	14.9%	±	8.1%	5.4%	±	5.2%	50.0%	±	11.4%	29.7%	±	10.4%	74
3’-gap both	9.5%	±	12.6%	0.0%	±	0.0%	57.1%	±	21.2%	33.3%	±	20.2%	21
5’-gap down	60.0%	±	42.9%	0.0%	±	0.0%	20.0%	±	35.1%	20.0%	±	35.1%	5
5’-gap up	48.7%	±	15.7%	2.6%	±	5.0%	38.5%	±	15.3%	10.3%	±	9.5%	39
5’-gap both	29.4%	±	21.7%	0.0%	±	0.0%	41.2%	±	23.4%	29.4%	±	21.7%	17

Table S11: Count of coilable and non-coilable tethers before and after RNase A treatment

	coilable before	Still coilable after	Lost bead after	Non-coilable after
With RNase A	31	0	6	25
Without ATP	16	12	4	0
Without RNase A	21	13	7	1
